# Chemical Profiling of *Gmelina philippensis* Cham. Leaf Extract and Its Antioxidant and Anti-Cholinesterase Properties

**DOI:** 10.3390/plants14223494

**Published:** 2025-11-16

**Authors:** Shaza H. Aly, Gyu Sung Lee, Yoon Seo Jang, Shaimaa Fayez, Ki Hyun Kim, Chung Sub Kim, Mohamed El-Shazly

**Affiliations:** 1 Department of Pharmacognosy, Faculty of Pharmacy, Badr University in Cairo (BUC), Cairo 11829, Egypt; shaza.husseiny@buc.edu.eg; 2 Department of Biopharmaceutical Convergence, Sungkyunkwan University, Suwon 16419, Republic of Korea; dlrbtjd36@skku.edu; 3 School of Pharmacy, Sungkyunkwan University, Suwon 16419, Republic of Korea; bbj0423@skku.edu; 4 Department of Pharmacognosy, Faculty of Pharmacy, Ain-Shams University, Cairo 11566, Egypt; shaimaa_fayez@pharma.asu.edu.eg; 5 Department of Biohealth Regulatory Science, Sungkyunkwan University, Suwon 16419, Republic of Korea; 6 Graduate Institute of Natural Products, College of Pharmacy, Kaohsiung Medical University, Kaohsiung 80708, Taiwan

**Keywords:** *Gmelina philippensis*, LC–MS/MS, Alzheimer’s disease, iridoid glycosides, antioxidant, acetylcholinesterase inhibition

## Abstract

*Gmelina philippensis* Cham. (Lamiaceae) is a traditionally valued medicinal plant with unexplored potential for the management of neurodegenerative disorders. In the present study, the phytochemical profile of its methanolic leaf extract was comprehensively characterized using untargeted liquid chromatography–tandem mass spectrometry metabolomics (LC–MS/MS) and molecular networking. In addition, the extract was evaluated for its antioxidant and cholinesterase inhibitory activities relevant to Alzheimer’s disease (AD). Metabolite profiling led to the annotation of 27 compounds, with a predominance of flavonoids and iridoid glycosides unique to the genus *Gmelina*, along with phenolic acids, lipids, and other minor compounds. The extract exhibited potent in vitro antioxidant activity, with an IC_50_ of 7.49 ± 0.002 μg/mL in the DPPH assay and 639.63 ± 0.814 μg AAE/mg in the FRAP assay. Notably, the extract showed significant inhibitory activity against acetylcholinesterase and butyrylcholinesterase, with an IC_50_ of 4.87 ± 0.16 and 40.99 ± 0.03 μg/mL, respectively. Molecular networking further supported the metabolite annotation and highlighted clusters of bioactive iridoids and flavonoids. Overall, these findings highlight that *G. philippensis* as a rich source of multi-target bioactive compounds, supporting that the extract has good anti-acetylcholinesterase activity comparable to the rivastigmine that used in neurodegenerative disease. This study provides a promising foundation for the development of novel therapeutic approaches targeting neurodegenerative diseases.

## 1. Introduction

Secondary metabolites derived from natural sources, particularly from plants, have long been recognized as important contributors to the treatment and prevention of various diseases [1]. These compounds play a pivotal role in combating complex health disorders, including oxidative stress, inflammatory conditions, wound healing, diabetes, and neurodegenerative diseases [2]. Among them, plant polyphenols are prominent natural antioxidants capable of mitigating oxidative damage associated with several pathological conditions such as cancer, liver and cardiovascular diseases, neurodegeneration, aging-related disorders, and diabetes [3,4]. This growing evidence reinforces the global interest in exploring natural products for the development of novel and effective therapeutic agents.

The genus *Gmelina* (Lamiaceae; formerly Verbenaceae) comprises approximately 40 species. Among these, *Gmelina philippensis* Cham., commonly referred to as Parrot’s Peak, is a perennial shrub widely cultivated for its ornamental value due to its distinctive golden-yellow inflorescence [5]. *G. philippensis* holds a longstanding history of ethnomedicinal use across several Asian countries, including Cambodia, Pakistan, and the Philippines, where it is traditionally employed to improve maternal and fetal health, treat gastrointestinal disorders, foot eczema, cough, and also used as a purgative and for managing joint and nerve-related conditions [6,7].

Previous phytochemical studies on the aerial parts of *G. philippensis* have consistently revealed its richness in iridoid glycosides—particularly rhamnopyranosylcatalpol esters—as well as flavonoids, terpenoids and fatty acids [6,7]. Although limited, pharmacological studies have reported antioxidant, antidiabetic, and enzyme inhibitory activities associated with *G. philippensis* extracts [6,7,8]. Furthermore, the ethanolic extract of its aerial parts has shown notable cytotoxic effects against hepatocellular carcinoma (HepG2) cell lines [7]. Network pharmacology-guided identification of *G. arborea* suggests that several phytochemicals can modulate various targets associated with AD pathology, such as amyloid-beta aggregation, tau hyperphosphorylation, oxidative stress, and neuroinflammation [9]. Also, *G. asiatica* showed neuroprotection properties in an in silico study [10].

Building on these findings, the present study aims to comprehensively characterize the phytochemical composition of the methanolic extract of *G. philippensis* using liquid chromatography–tandem mass spectrometry (LC–MS/MS) and to evaluate its antioxidant capacity and cholinesterase inhibitory potential through in vitro 2,2-diphenyl-1-picrylhydrazyl (DPPH), ferric reducing antioxidant power (FRAP), acetylcholinesterase (AChE), and butyrylcholinesterase (BChE) assays.

## 2. Results and Discussion

### 2.1. Untargeted Metabolome Analysis

A comprehensive metabolomic profiling of the methanolic extract of *Gmelina philippensis* leaves was carried out using untargeted liquid chromatography–tandem mass spectrometry (LC–MS/MS) in both positive and negative ionization modes (For chromatograms see Figure S1). A total of 27 metabolites were annotated based on the alignment of retention times, accurate mass values, and MS/MS fragmentation patterns with previously reported data [11,12].

**Figure 1:**
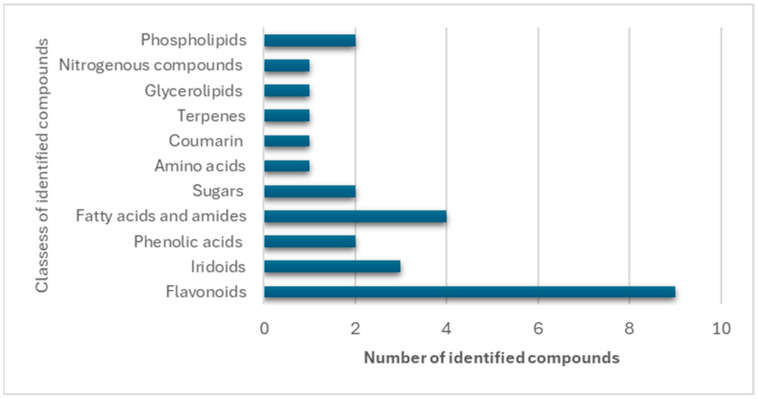
Different classes of metabolites identified in the methanol extract of *G. philippensis* leaves.

The identified metabolites were grouped into distinct phytochemical classes according to their polarity and elution order. Compounds eluted in the sequence of decreasing polarity, starting with phenolic acids, followed by flavonoid and iridoid glycosides, their aglycones, and finally fatty acids and phospholipids. Among these, flavonoids constituted the most abundant class, accounting for nine identified compounds. Overall, the detected metabolites comprised nine flavonoids, three iridoid glycosides, two phenolic acids, four fatty acids and fatty acid amides, two phospholipids, and miscellaneous compounds, including sugars, a diterpene, and a coumarin derivative (Table 1 and Figure 1).

#### 2.1.1. Flavonoids

Flavonoids were the predominant class of metabolites detected in the methanolic extract of *G. philippensis*, with a particular abundance of flavone and flavonol glycosides. Notable representatives included derivatives of isovitexin, rhoifolin, quercetin, and kaempferol, which corresponded to nine distinct chromatographic peaks (peaks **8** and **10**–**17**).

The identification of these flavonoid glycosides was primarily based on two characteristic MS/MS fragmentation features: (i) the neutral loss of sugar moieties, indicative of glycosidic cleavage, and (ii) the Retro-Diels–Alder (RDA) fragmentation pattern of the aglycone. The aglycone ion, often observed as the base peak following deglycosylation, was confirmed by its accurate mass corresponding to the expected loss of the sugar unit [13].

Peak **8** (*m*/*z* 563.1408; [M−H]^−^) exhibited a characteristic fragmentation pattern consistent with a flavone di-*C*-hexoside. Diagnostic fragment ions were observed at *m*/*z* 473.1100 ([M−H−90]^−^) and 443.1005 ([M−H−120]^−^), corresponding to typical cross-ring cleavages of sugar moieties. Additional fragment ions at *m*/*z* 353.0678 and 383.0779, assigned as [Aglycone+83]^−^ and [Aglycone+113]^−^, respectively, indicated the presence of a di-*C*-glycoside structure retaining partial sugar residues on the aglycone, as previously described by Farag et al. [14]. Accordingly, peak **8** was identified as apigenin 6-*C*-arabinoside-8-*C*-glucoside (isoschaftoside).

Peak **10** (*m*/*z* 433.1127; [M+H]^+^) was annotated as isovitexin, a mono-*C*-glycosyl flavone. Its MS/MS spectrum showed a prominent fragment at *m*/*z* 313.0706 ([M−120]^−^), indicative of sugar cleavage specific to *C*-hexosides.

Peak **14** (*m*/*z* 577.1559; [M−H]^−^) was assigned as apigenin 7-*O*-neohesperidoside (rhoifolin), based on its fragment ions at *m*/*z* 413.0839 ([M−H−164]^−^) and 269.0441 ([M−H−308]^−^), corresponding to the sequential loss of rhamnose moiety and H_2_O (−164 Da) and hexosyl and rhamnose moieties (−308 Da), consistent with the formation of apigenin aglycone [15].

Several apigenin glycosides, including vicenin-II, rhoifolin, and isorhoifolin, have been previously isolated from the butanol fraction of the aerial parts of *G. philippensis* [7].

The presence of a kaempferol aglycone fragment ion (*m*/*z* 286; C_15_H_10_O_6_) was observed in peaks **13**, **16**, and **17**, supporting their structural annotation as kaempferol glycosides. Specifically, peak **13** (*m*/*z* 447.0931; [M−H]^−^) showed a prominent fragment ion at *m*/*z* 284.0310, corresponding to the neutral loss of a hexose moiety (−162 Da), consistent with its identification of peak **13** as kaempferol 3-*O*-glucoside (astragalin). Peak **16** (*m*/*z* 417.0827; [M−H]^−^), showed a major fragment ion at *m*/*z* 284.0323, indicate of pentose loss (−132 Da). confirming its identity as kaempferol-3-*O*-arabinoside (juglalin) [16]. In the positive ionization mode, peak **17** (*m*/*z* 565.1546; [M+H]^+^) was identified as kaempferol 3-*O*-α-l-arabinopyranosyl-7-*O*-α-l-rhamnopyranoside, based on its molecular formula and characteristic kaempferol-based structure.

Peak **11** (*m*/*z* 463.0871; [M−H]^−^) and peak **12** (*m*/*z* 433.0776; [M−H]^−^) yield a quercetin aglycone ion (*m*/*z* 302; C_15_H_10_O_7_), supporting their annotation as quercetin-3-*O*-galactoside and quercetin-3-*O*-xyloside, respectively. Both compounds showed base fragment ions at *m*/*z* 300.0264 and 300.0262, resulting from the neutral loss of hexose (−162 Da) and pentose (−132 Da) moieties, respectively.

Additionally, peak **15** (*m*/*z* 287.0543; [M+H]^+^) was identified as luteolin, representing a free flavone.

These findings are consistent with previous phytochemical reports on *G. arborea*, in which kaempferol, quercetin, and luteolin were isolated from various plant organs either in glycosylated or aglycone forms [17].

#### 2.1.2. Iridoid Glycosides

LC–MS/MS analysis enabled the identification of three iridoid glycosides characteristic of the genus *Gmelina*, namely 3″-*O*-caffeoyl-6-*O*-rhamnopyranosylcatalpol (peak **7**), saccatoside (peak **9**), and gmelinoside N (peak **19**).

Peak **7** (*m*/*z* 669.2027; [M−H]^−^) was annotated as 3″-*O*-caffeoyl-6-*O*-rhamnopyranosylcatalpol. Its MS/MS spectrum exhibited a diagnostic fragment ion at *m*/*z* 179.0334, corresponding to the caffeoyl moiety (dihydroxycinnamoyl group), followed by a secondary fragment at *m*/*z* 161.0232, resulting from the neutral loss of a H_2_O (−18 Da) from the caffeoyl group, supporting the proposed structure.

Peak **9** (*m*/*z* 653.2088; [M−H]^−^) was identified as saccatoside, also known as 6-*O*-α-(2″-*O*-*p*-coumaroyl) rhamnopyranosylcatalpol. The MS/MS fragmentation revealed a prominent fragment ion at *m*/*z* 291.0879 attributed to the catalpol moiety, as well as an ion at *m*/*z* 163.0404 corresponding to the *p*-coumaroyl group. A subsequent fragment at *m*/*z* 145.0299, resulting from H_2_O loss (−18 Da) from the coumaroyl residue, further supported the identification. These findings are in agreement with previous reports of saccatoside in the leaves of *G. arborea* [17,18].

Peak **19** (*m*/*z* 799.2465; [M−H]^−^) was identified as gmelinoside N. This compound is structurally known as 6-*O*-α-l-(2″,3″-di-*O*-trans-*p*-hydroxycinnamoyl)rhamnopyranosylcatalpol. Fragmentation analysis revealed an ion at *m*/*z* 653.2079, corresponding to the loss of a rhamnose unit (−146 Da). Additional fragment ions at *m*/*z* 163.0401 and 145.0297 were assigned to the *p*-hydroxycinnamoyl moiety and its H_2_O loss (−18 Da), respectively. A fragment ion at *m*/*z* 437.1234 was also detected, indicating the presence of the catalpol skeleton after cleavage of sugar and cinnamoyl groups. This compound was previously isolated from the flowers of *G. arborea* [19].

#### 2.1.3. Phenolic Acids

Two phenolic glycosides were identified in the methanolic extract. Peak **5** (*m*/*z* 325.0925; [M−H]^−^) was annotated as a *p*-coumaric acid glucoside, based on the presence of diagnostic fragment ions at *m*/*z* 163.0382 and 119.0491, corresponding to *p*-coumaric acid. A base peak at *m*/*z* 145.0284, resulting from the H_2_O loss (−18 Da), further supported this identification [20].

Peak **6** (*m*/*z* 309.0967; [M−H_2_O+H]^+^) was assigned as trans-β-d-glucosyl-2-hydroxycinnamate. Its MS/MS spectrum showed a base peak at *m*/*z* 147.0428, arising from the loss of a glucose moiety (−162 Da), a fragmentation pattern commonly observed for hydroxycinnamate glycosides.

These phenolic acids and their glycosylated derivatives have previously been reported in *G. arborea* [21,22]. Moreover *p*-coumaric and cinnamic acid derivatives are known to be common structural components of iridoid glycosides in various *Gmelina* species [17,23].

#### 2.1.4. Lipids

Fatty acids and fatty acid amides were annotated in peaks **20**, **22**, **26**, and **27**. Peak **20** (*m*/*z* 329.2330; [M−H]^−^) was identified as 9,12,13-trihydroxy-10-*E*-octadecenoic acid. peak **22** (*m*/*z* 279.2317; [M+H]^+^) was assigned as α-linolenic acid. Peaks **26** (*m/z* 322.2730; [M+H]^+^) and **27** (*m*/*z* 324.2887; [M+H]^+^) were annotated as fatty acid ethanolamides, specifically α-linolenoyl ethanolamide and linoleoyl ethanolamide, respectively, based on accurate mass measurements and characteristic MS/MS fragmentation patterns.

In addition, two phospholipids were characterized in peaks **23** (*m*/*z* 452.2784; [M−H]^−^) and **25** (*m*/*z* 496.3392; [M+H]^+^) based on their exact masses and fragmentation patterns. Peak **24** (*m*/*z* 353.2685; [M+H]^+^) was assigned as 1-monolinolenin.

The presence of similar fatty acids and related lipids has previously been reported in the leaves of *G. asiatica* L. [24] and in the seeds of *G. arborea* [17,25,26].

#### 2.1.5. Miscellaneous

LC–MS/MS analysis also revealed the presence of several miscellaneous compounds. Peak **18** (*m*/*z* 277.1064; [M+H]^+^) was identified as the coumarin derivative murrangatin, exhibiting characteristic base fragment ion at *m*/*z* 131.0487 [27]. Additional compound classes identified in the extract included terpenoids, sulfonic acids, sugars, as well as carboxylic and amino acids, as summarized in Table 1.

Overall, the LC–MS/MS profiling of the methanolic extract of *G. philippensis* revealed a chemically diverse and structurally complex metabolite profile that substantiates its traditional medicinal applications. The detection of multiple compound classes—such as iridoid glycosides, flavonoids, terpenoids, phenolic acids, and structurally diverse minor constituents—emphasizes the rich phytochemical composition of this species.

This comprehensive metabolomic characterization provides a solid foundation for future pharmacological investigations and highlights the potential of *G. philippensis* as a valuable source of bioactive natural products with therapeutic relevance.

### 2.2. LC–MS/MS Analysis and Molecular Networking

**Figure 2:**
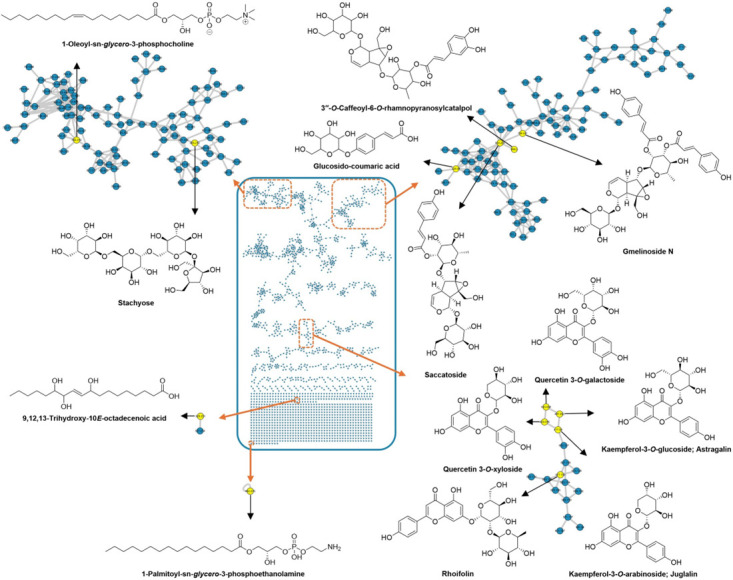
Untargeted analysis of the methanol extract of *G. philippensis* leaves using MS/MS molecular networking (negative mode).

A metabolomics investigation guided by LC–MS/MS analysis was conducted to elucidate the chemical complexity of the methanolic extract of *G. philippensis* leaves. For metabolite annotation and structural classification, MS/MS-based molecular networking was employed using the Global Natural Products Social Molecular Networking (GNPS) platform. Spectral library matching facilitated dereplication of known metabolites, while clustering based on fragment ion similarity revealed relationships among structurally related compound families. The constructed molecular network—based on data acquired in negative ionization mode—consisted of 2079 nodes, grouped into 157 clusters, including 737 self-looped nodes (Figure 2). In this network, nodes represent precursor ions, and edges indicate the mass differences between connected features. GNPS-based molecular networking enabled the dereplication of several secondary metabolite classes, including iridoid glycosides (e.g., gmelinoside N, saccatoside, and caffeoyl-rhamnopyranosyl catalpol), cinnamic acid derivatives (e.g., *p*-coumaric acid glucoside), flavonoids (e.g., astragalin, rhoifolin, juglalin, isoschaftoside, luteolin, and quercetin glucoside), as well as benzene sulfonic acids, phospholipids, and unsaturated fatty acids.

### 2.3. Assessment of the Antioxidant Activity Using DPPH and ABTS Assays

**Figure 3:**
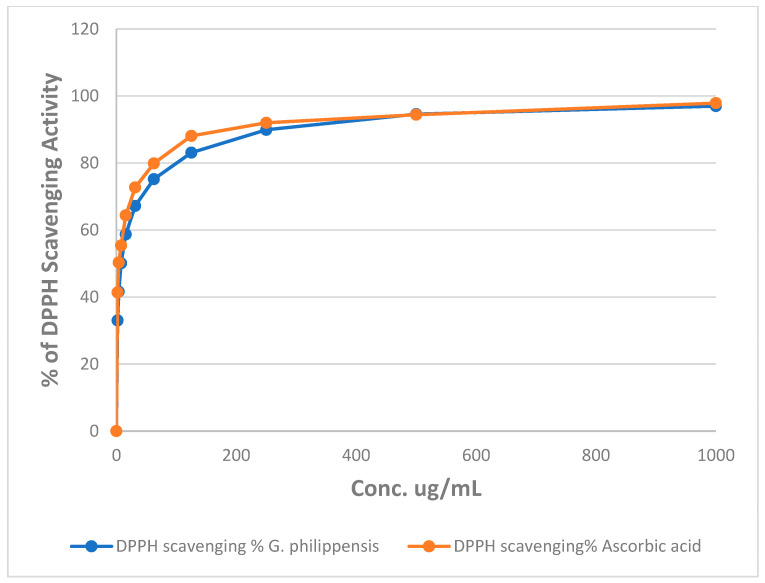
Changes in the % of DPPH assay of different concentrations of the methanol extract of *G. philippensis* and ascorbic acid (positive control).

The percentages of DPPH radical scavenging activity of different concentrations of the methanol extract of *G. philippensis* are illustrated in Figure 3. The results revealed that *G. philippensis* showed an IC_50_ of 7.49 ± 0.002 μg/mL as compared to ascorbic acid with an IC_50_ of 3.6 ± 0.001 μg/mL, revealing a promising antioxidant potential. Moreover, in FRAP assay, the methanol extract of *G. philippensis* showed 639.63 ± 0.814 µg equivalent ascorbic acid equivalent (AAE) /mg of extract. From the previously reported results, leaves and flowers of *G. philippensis* from Dhaka, Bangladesh, showed significant variation in antioxidant activity depending on the solvent used for fractionation. DPPH IC_50_ values ranged from 9.75 to 35.25 μg/mL, indicating varying degrees of free-radical scavenging capacity [8]. Another study revealed the antioxidant activity of the *n*-hexane extract from *G. philippensis* leaves by different assays as DPPH and ABTS, cupric reducing antioxidant capacity (CUPRAC) and metal chelating ability (MCA) assays [6]. A major constituent in the extract is the iridoid glycosides which have been reported for their antioxidant potential [28].

### 2.4. Assessment of the In Vitro Inhibition of Acetylcholinesterase (AChE) and Butyrylcholinesterase (BChE) Enzyme Activities

**Figure 4:**
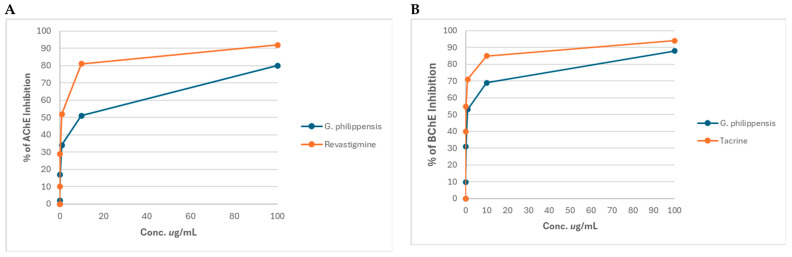
Acetylcholinesterase (**A**) and Butyrylcholinesterase (**B**) inhibitory activity of the methanol extract of *G. philippensis* leaves.

The percentage of AChE and BChE enzyme activities after incubation with different concentrations of the methanol extract of *G. philippensis* are illustrated in Figure 4A,B. The extract exhibited AChE inhibitory activity with an IC_50_ of 4.869 ± 0.16 μg/mL, compared to the standard rivastigmine (0.73 ± 0.025 μg/mL). It also showed BChE inhibitory effects with an IC_50_ of 40.985 ± 0.034 μg/mL compared to the standard tacrine (0.044 ± 0.002 μg/mL). At a concentration of 10 μg/mL, *G. philippensis* methanol extract inhibited butyrylcholinesterase by 69%, compared to tacrine (85%) and inhibited acetylcholinesterase by 51%, compared to rivastigmine (81%), revealing a promising anti-Alzheimer’s potential. This finding is corroborated by a previous study that revealed that the flower extract demonstrated the most promising anti-Alzheimer activity through AChE inhibition with an IC_50_ of 89.07 ± 5.14 μg/mL, followed by leaves (IC_50_ = 137.22 ± 7.39 μg/mL). The prevalence of glycosides (gmelinosides) is particularly noteworthy, as these compounds represent a unique chemical class with potential neuroprotection [29,30,31].

## 3. Materials and Methods

### 3.1. Plant Material Collection and Authentication

Leaves of *Gmelina philippensis* were collected in March 2021 from El Orman Botanical Garden, Giza, Egypt. Dr. Mohammed El-Gebaly (National Research Centre, Giza, Egypt) authenticated the plant. A voucher specimen (BUC-PHG-GP-12) was deposited at Badr University in Cairo’s Pharmacognosy Department.

### 3.2. Extraction Procedure

Dried leaves (10 g) of *G. philippensis* underwent sequential solvent extraction. First, defatting was performed via maceration in *n*-hexane for three days. After filtration, the solvent was evaporated using a Rotavap (40 °C), yielding 294 mg of hexane extract. The residual plant material was then extracted with 100% methanol, yielding 2.32 g of dried methanolic extract. This extract was stored refrigerated for subsequent analysis [11].

### 3.3. HPLC-MS Analysis

Separation was performed on a Waters ACQUITY UPLC^®^ BEH C18 column (150 × 2.1 mm i.d., 1.7 μm) maintained at 30 °C [32,33]. A gradient elution program was employed using mobile phase A (water with 0.1% formic acid) and B (acetonitrile) as follows: 15% B (0.00–1.00 min); linear increase to 100% B (1.00–16.00 min); hold at 100% B (16.00–18.00 min); rapid return to 15% B (18.00–18.01 min); hold at 15% B (18.01–20.00 min). The post-time was 2.00 min for column re-equilibration. The extract was first dissolved in 100% methanol, and then diluted with an acetonitrile-water (1:1, *v*/*v*) mixture to a final concentration of 2000 ppm, and an aliquot (3 µL) was injected. MS/MS analysis utilized both positive and negative electrospray ionization (ESI) modes. Key parameters included: gas temperature 320 °C; gas flow 8 L/min; nebulizer pressure 35 psi; sheath gas temperature 350 °C; sheath gas flow 11 L/min; capillary voltage 3500 V; nozzle voltage 1000 V; fragmentor voltage 100 V. Full-scan MS spectra (100–1700 *m*/*z*) were acquired at 3 spectra/s (333 ms/spectrum), while MS/MS spectra (100–1700 *m*/*z*) were acquired at 2 spectra/s (500 ms/spectrum). Collision energy used a linear ramp (offset 16.6 eV; slope 3.3 eV per 100 *m*/*z*). Real-time mass calibration employed internal references purine and HP-0921, providing lock masses at *m*/*z* 121.0508, 322.0481, 922.0097 (positive mode) and *m*/*z* 112.9855, 119.0363, 966.0007, 980.0163 (negative mode).

### 3.4. GNPS-Based Molecular Networking

The raw mass data files obtained from the untargeted LC-MS analysis of the methanol extract of *G. philippensis* leaves were converted to mzML using MSConvert tool from ProteoWizard version 3.0.22288 (http://proteowizard.sourceforge.net, accessed on 1 March 2024). The chromatogram was centroided and then uploaded to the GNPS platform (http://gnps.ucsd.edu, accessed on 1 march 2024) to construct a network in positive and negative modes. Clustering and spectral library search were performed based on the similarity in MS/MS fragment ions whose precursor and fragment ion mass tolerance were set at 0.02 Da each. The advanced network options were as follows: Minimum pairs cosine of 0.6, network TopK of 10, maximum connected component size of 100, maximum matched fragment ions of 3, and minimum cluster size of 2. For the library search, a minimum of 3 matching peaks with a score threshold of 0.6 was selected. All other parameters were kept at default values. The molecular networks generated in GNPS were exported to Cytoscape (version 3.9.1) in ‘graphml’ format to enable customized visualization and further analysis [34].

### 3.5. Evaluation of Antioxidant Activity by DPPH Radical Scavenging Method

The free-radical scavenging activity of the extract was evaluated using the DPPH method. Briefly, different concentrations of the extract (1.95–1000 µg/mL) were prepared in ethanol and were mixed with 0.1 mM ethanolic DPPH solution (1 mL extract + 3 mL DPPH). After vigorous shaking and 30 min incubation at room temperature, absorbance was measured at 517 nm using a UV-Vis spectrophotometer. Ascorbic acid served as the standard reference, and experiments were performed in triplicate. The percentage inhibition was calculated as [(A_0_ − A_1_)/A_0_] × 100, where A_0_ is the control (DPPH + ethanol) absorbance and A_1_ is the test/standard absorbance. Lower absorbance indicated higher activity. The IC_50_ value (concentration inhibiting 50% of DPPH radicals) was determined from a log dose inhibition curve [35].

### 3.6. Ferric Reducing Antioxidant Power (FRAP) Assay

The total reducing power (TRP, equivalent to FRAP) of the extract was assessed using a modified microplate adaptation [36] of the potassium ferricyanide-trichloroacetic acid method [37]. In brief, 40 µL sample was mixed with 50 µL sodium phosphate buffer (0.2 mol/L), 50 µL potassium ferricyanide (1%), and 50 µL trichloroacetic acid (10%). After centrifugation (3000 rpm, 10 min), 166.66 µL of supernatant was transferred to a 96-well plate, reacted with 33.3 µL ferric chloride (1%), and absorbance was measured at 630 nm using a Biotek ELX800 microplate reader. DMSO and ascorbic acid (1 mg/mL) served as negative and positive controls, respectively. Results are expressed as ascorbic acid equivalents (AAE) per mg of extract.

### 3.7. In Vitro Assay for Acetylcholinesterase (AChE) and Butyrylcholinesterase (BChE) inhibition

The acetylcholinesterase (AChE) and Butyrylcholinesterase (BChE) inhibitory activity of the extract was assessed using Bio-vision’s AChE Inhibitor Screening Kit (Cat. #K197-100, Bio Vision, Egypt), and BChE Activity Assay Kit (Colorimetric) (LS-K17-100, LSBio, LifeSpan Bio Sciences Inc., Newark, CA, USA) following a published method [38,39]. Briefly, the extract was dissolved in DMSO (Merck), to prepare the extract in different concentrations. The sample mixture was further diluted with the assay buffer then 10 μL of diluted test inhibitors were transferred into the appropriate wells of a 96-well plate. The reference inhibitors used in the assay were rivastigmine and tacrine. Acetylthiocholine iodide served as the colorimetric substrate in the reaction, while 5,5′-dithio-bis(2-nitrobenzoic acid) (DTNB) was utilized to measure cholinesterase activity. The absorbance (OD) was measured at 412 nm. The concentration of the sample required to inhibit 50% of the enzyme activity (IC_50_) was determined by plotting the percentage of enzyme inhibition against the sample concentrations.

## 4. Conclusions

This study provides the first comprehensive phytochemical and biological assessment of *G. philippensis* leaf methanol extract targeting Alzheimer’s disease (AD) pathology. Untargeted LC–MS/MS analysis identified 27 metabolites, revealing a complex profile rich in flavonoids and iridoid glycosides, alongside phenolic acids, lipids, and phospholipids. Molecular networking further corroborated these annotations, emphasizing structural clusters associated with bioactivity. In vitro evaluations demonstrated remarkable antioxidant capacity, evidenced by low DPPH IC_50_ (7.49 μg/mL) and high FRAP value (639.63 μg AAE/mg), attributable to the abundance of polyphenols and iridoids. Crucially, the extract exhibited potent dual cholinesterase inhibition, with an IC_50_ of 4.87 μg/mL (AChE) and 40.99 μg/mL (BChE), suggesting its ability to enhance cholinergic neurotransmission—a key therapeutic target in AD. The presence of iridoids and flavonoids aligns with these activities, as both classes are known for neuroprotective properties. These findings validate *G. philippensis*’s traditional medicinal uses and position it as a promising source of multi-target agents against AD. However, further in vivo studies and detailed mechanistic investigations are required to validate these results and advance the development of lead compounds for neurodegenerative therapies.

## Figures and Tables

**Table 1:** Annotated metabolites in the methanol extract of *G. philippensis* using LC–MS/MS analysis in positive and negative modes.

Peak No.	*t*_R_ ^a^ (min)	Annotated Compound	Calculated Mass (*m*/*z*)	Observed Mass (*m*/*z*)	Adduction	MS/MS ^b^	Δ ppm	Chemical Formula	Class	Confidence Level ^C^
1	0.89	Stachyose	665.2146	665.2151	[M−H]^−^	59.0142, 71.0139, 89.0245, 101.0243, 113.0246, 119.0353, 165.0406, 179.0562, **383.1192**	0.75	C_24_H_42_O_21_	Sugar	Level 2
2	0.91	Sucrose	365.1054	365.1051	[M+Na]^+^	157.0078, 185.0408, **203.0515**	−0.82	C_12_H_22_O_11_	Sugar	Level 2
3	0.93	Nicotinic acid	124.0393	124.0388	[M+H]^+^	53.0383, **78.0332**, 80.0489	−4.03	C_6_H_5_NO_2_	Pyridinecarboxylic acid	Level 2
4	0.94	L-Pipecolic acid	130.0863	130.0856	[M+H]^+^	56.0492, 77.0378, **84.0798**, 103.0531, 117.0560	−5.38	C_6_H_11_NO_2_	Amino acid	Level 2
5	1.90	*p*-Coumaric acid glucoside	325.0929	325.0925	[M−H]^−^	59.0135, 71.0132, 117.0336, 119.0491, **145.0284**, 161.0595, 163.0382	−1.23	C_15_H_18_O_8_	Phenolic acid	Level 2
6	2.29	*trans-*β-d-Glucosyl-2-hydroxycinnamate	309.0969	309.0967	[M−H_2_O+H]^+^	84.9588, 91.0535 119.0481, **147.0428**, 194.8577	−0.65	C_15_H_18_O_8_	Phenolic acid	Level 2
7	2.34	3″-*O*-Caffeoyl-6-*O*-rhamnopyranosylcatalpol	669.2036	669.2027	[M−H]^−^	85.0302, 116.9275, 133.0282, 135.0436, 145.0288, **161.0232**, 179.0334, 337.4182, 426.9881	−1.34	C_30_H_38_O_17_	Iridoid glycoside	Level 2
8	2.39	Apigenin 6-*C*-arabinoside 8-*C*-glucoside (Isoschaftoside)	563.1406	563.1408	[M−H]^−^	297.0784, 325.0715, **353.0678**, 365.0669, 383.0779, 395.0783, 413.0891, 425.0896, 443.1005, 473.1100	0.36	C_26_H_28_O_14_	Flavone glycoside	Level 2
9	3.42	Saccatoside	653.2087	653.2088	[M−H]^−^	119.0503, **145.0299**, 163.0404, 291.0879, 377.1249	0.15	C_30_H_38_O_16_	Iridoid glycoside	Level 2
10	3.89	Apigenin-6-*C*-glucoside (Isovitexin)	433.1129	433.1127	[M+H]^+^	147.0442, **283.0589**, 313.0706, 323.0905, 337.0710	−0.46	C_21_H_20_O_10_	Flavone glycoside	Level 2
11	4.32	Quercetin 3-*O*-galactoside	463.0882	463.0871	[M−H]^−^	59.0135, 77.0397, 121.0274, 135.0432, 145.0284, 151.0022, 178.9971, 243.0401, 255.0291, 271.0230, **300.0264**	−2.38	C_21_H_20_O_12_	Flavonol glycoside	Level 2
12	4.64	Quercetin 3-*O*-xyloside	433.0776	433.0776	[M−H]^−^	151.0030, 163.0031, 178.9974, 243.0278, 255.0293, 271.0239, 283.0243, **300.0262**	0.00	C_20_H_18_O_11_	Flavonol glycoside	Level 2
13	5.00	Kaempferol 3-*O*-glucoside (Astragalin)	447.0933	447.0931	[M−H]^−^	59.0131, 107.0128, 151.0012, 175.0368, 227.0331, 255.0282, **284.0310**	−0.45	C_21_H_20_O_11_	Flavonol glycoside	Level 2
14	5.03	Apigenin 7-*O*-neohesperidoside (Rhoifolin)	577.1563	577.1559	[M−H]^−^	163.0396, 205.0495, **269.0441**, 413.0839	−0.69	C_27_H_30_O_14_	Flavone glycoside	Level 2
15	5.35	Luteolin	287.0550	287.0543	[M+H]^+^	133.0633, **153.0180**, 161.0588, 241.0473, 269.0426	−2.44	C_15_H_10_O_6_	Flavone	Level 2
16	5.35	Kaempferol 3-*O*-arabinoside (Juglalin)	417.0827	417.0827	[M−H]^−^	68.9989, 107.0494, 149.0602, 227.0320, 255.0306, 257.0413, **284.0323**, 297.0764	0.00	C_20_H_18_O_10_	Flavonol glycoside	Level 2
17	5.33	Kaempferol 3-*O*-α-l-arabinopyranosyl-7-*O*-α-l-rhamnopyranoside	565.1552	565.1546	[M+H]^+^	85.0284, **287.0552**, 469.1318	−1.06	C_26_H_28_O_14_	Flavonol glycoside	Level 2
18	6.58	Murrangatin	277.1071	277.1064	[M+H]^+^	55.0545, 69.0334, 103.0537, **131.0487**, 172.9893	−2.53	C_15_H_16_O_5_	Coumarin	Level 2
19	7.29	Gmelinoside N (6-*O*-α-l-(2″, 3″-di*-O-trans*-*p*-hydroxycinnamoyl)rhamnopyranosylcatalpol)	799.2455	799.2465	[M−H]^−^	59.0137, 119.0501, 135.0446, **145.0297**, 163.0401, 187.0405, 205.0520, 291.0864, 359.1142, 377.1237, 437.1234, 455.1332, 523.1624, 653.2079	1.25	C_39_H_44_O_18_	Iridoid glycoside	Level 2
20	8.01	9,12,13-Trihydroxy-10*E*-octadecenoic acid	329.2333	329.233	[M−H]^−^	57.0349, 59.0127, 71.0510, 99.0810, 127.1122, 139.1122, 165.1275, 171.1020, 183.1389, 193.1218, 209.1194, **211.1339**, 229.1437	−0.91	C_18_H_34_O_5_	Fatty acid	Level 2
21	10.80	Gelomulide N	415.2115	415.2111	[M−H_2_O+H]^+^	91.0536, 107.0856, **119.0856**, 133.0645	−0.96	C_24_H_32_O_7_	Diterpenoids	Level 2
22	12.66	α-Linolenic acid	279.2319	279.2317	[M+H]^+^	**57.0696**, 67.0537, 71.0851, 81.0694, 95.0845, 183.1354	−0.72	C_18_H_30_O_2_	Fatty acid	Level 2
23	13.17	1-Palmitoyl-2-hydroxy-sn-*glycero*-3-phosphoethanolamine	452.2783	452.2784	[M−H]^−^	57.0341, 59.0129, 71.0149, 75.0085, 78.9592, 87.0074, 140.0107, 166.9247, 177.0393, 196.0364, **255.2324**, 277.2187, 390.7082	0.22	C_21_H_44_NO_7_P	Phospholipid	Level 2
24	14.51	1-Monolinolenin	353.2686	353.2685	[M+H]^+^	55.0545, 67.0540, **81.0695**, 95.0850, 107.0855, 121.1007, 135.1162, 261.2216, 300.1405	−0.28	C_21_H_36_O_4_	Glycerolipids	Level 2
25	13.20	Lysophosphatidylcholine (16:0)	496.3398	496.3392	[M+H]^+^	60.0807, 86.0962, 104.1067, 124.9995, **184.0728**, 313.2718	−1.21	C_24_H_50_NO_7_P	Phospholipid	Level 2
26	13.38	α-Linolenoyl ethanolamide	322.2741	322.273	[M+H]^+^	55.0542, **62.0596**, 67.0534, 81.0694, 93.0697, 107.0847, 154.1217, 179.1043, 309.0737	−3.41	C_20_H_35_NO_2_	Fatty amides	Level 2
27	14.47	Linoleoyl ethanolamide	324.2897	324.2887	[M+H]^+^	57.0696, **62.0597**, 67.0539, 81.0695, 95.0853, 109.0995, 238.8297, 290.1763	−3.08	C_20_H_37_NO_2_	Fatty amides	Level 2

^a^ Retention time ^b^ Fragment ions shown in bold represent base peak in the MS/MS spectra. ^C^ Confidence levels based on Metabolomics Standards Initiative guidelines: Level 1 = confirmed by standards; Level 2 = MS/MS library match; Level 3 = compound class match.

## Data Availability

The original contributions presented in this study are included in the article/Supplementary Material. Further inquiries can be directed to the corresponding author.

## Supplementary Materials

The following supporting information can be downloaded at: https://www.mdpi.com/article/10.3390/plants14223494/s1, Figure S1. Total ion chromatogram (TIC) of the methanol extract of *G. philippensis* leaves in positive ion mode (**A**) and negative ion mode (**B**).
