# Supplementary material for: Chemical Profiling of Gmelina philippensis Cham. Leaf Extract and Its Antioxidant and Anti-Cholinesterase Properties
Source: Plants (Basel). 2025 Nov 16;14(22):3494. doi: 10.3390/plants14223494 (PMC12656140; doi:10.3390/plants14223494)
Supplement: Supplementary file 1 [file plants-14-03494-s001.zip › plants-3915063-supplementary.pdf]

Supplementary file

Chemical Profiling of Gmelina philippensis Cham. Leaf Extract and Its Antioxidant and Anti-Cholinesterase Properties

Shaza H. Aly [1,†], Gyu Sung Lee [2,†], Yoon Seo Jang [3,†], Shaimaa Fayez [4,5], Ki Hyun Kim [3,*], Chung Sub Kim [2,3,4,*] and Mohamed El-Shazly [5,6,*]

1 Department of Pharmacognosy, Faculty of Pharmacy, Badr University in Cairo (BUC), Cairo 11829, Egypt; shaza.husseiny@buc.edu.eg
2 Department of Biopharmaceutical Convergence, Sungkyunkwan University, Suwon 16419, Republic of Korea; dlrbtjd36@skku.edu
3 School of Pharmacy, Sungkyunkwan University, Suwon 16419, Republic of Korea; bbj0423@skku.edu
4 Department of Biohealth Regulatory Science, Sungkyunkwan University, Suwon 16419, Republic of Korea; shaimaa_fayez@pharma.asu.edu.eg
5 Department of Pharmacognosy, Faculty of Pharmacy, Ain-Shams University, Cairo 11566, Egypt
6 Graduate Institute of Natural Products, College of Pharmacy, Kaohsiung Medical University, Kaohsiung 80708, Taiwan
* Correspondence: khkim83@skku.edu (K.H.K.); chungsub.kim@skku.edu (C.S.K.); cmohamed.elshazly@pharma.asu.edu.eg (M.E.-S.)

† These authors contributed equally to this work.

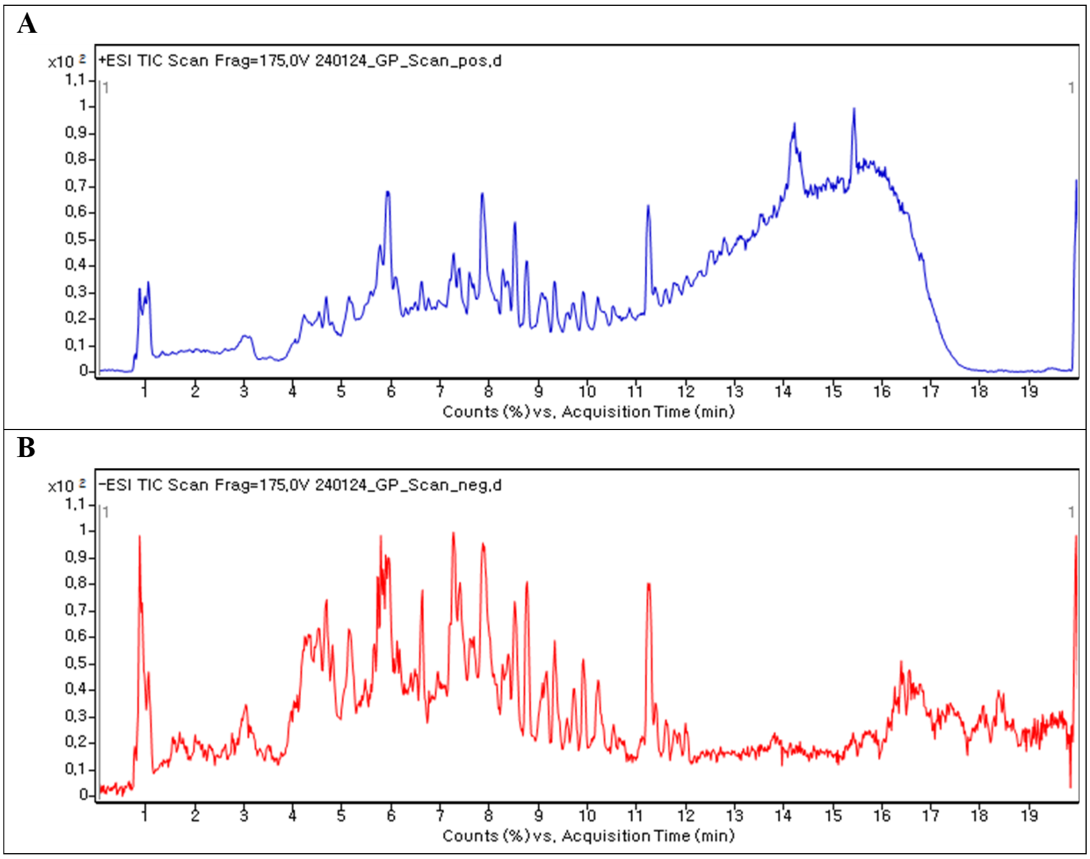

Figure S1. Total ion chromatogram (TIC) of the methanol extract of *G. philippensis* leaves in positive ion mode (**A**) and negative ion mode (**B**)
